# Completeness of birth registration in Brazil: an overview of methods and data sources

**DOI:** 10.1186/s41118-018-0035-9

**Published:** 2018-08-09

**Authors:** Everton E. C. Lima, Bernardo Lanza Queiroz, Krystof Zeman

**Affiliations:** 1 College of Philosophy and Human Sciences (IFCH), Universidade Estadual de Campinas, Núcleo de Estudos de População “Elza Berquó”, Av. Albert Einstein, 1300, Cidade Universitária Zeferino Vaz, Campinas, SP 13081-970 Brazil; 2 Núcleo de Estudos Populacionais (NEPO), Unicamp, Campinas, Brazil; 3 Universidade Federal de Minas Gerais and Cedeplar, Belo Horizonte, Brazil; 4 Wittgenstein Centre for Demography and Global Human Capital (IIASA, VID/ÖAW, WU), Vienna Institute of Demography (Austrian Academy of Sciences), Vienna, Austria

**Keywords:** Fertility, Vital registration system, Population censuses, Indirect demographic methods, Brazil

## Abstract

We provide an analysis of the main sources of data used to estimate fertility schedules in developing countries, giving special attention to Brazil. In addition to the brief history of various data sources, we present several indirect demographic methods, commonly used to estimate fertility and assess the quality of data. From the methods used, the Synthetic Relational Gompertz model gives the most robust estimates of fertility, independent of the data source considered. We conclude that different demographic data sources and methods generate differing estimates of fertility and that the country should invest in quality of birth statistics.

**Electronic supplementary material:**

The online version of this article (10.1186/s41118-018-0035-9) contains supplementary material, which is available to authorized users.

## Introduction

In less developed and developing countries, there is often a common concern among demographers about data quality, especially concerning the vital registration systems, and a strong consensus about the necessity to improve vital statistics (Mikkelsen et al. 2015; AbouZahr et al. 2015). This concern is understandable as many countries in the world, and especially in Latin America, are still suffering from a considerable amount of data problems (Faijer 1994; Duryea et al. 2006; Alkema et al. 2012; Hunter and Sugiyama 2018).

Having high-quality vital registration systems is one of the targets of sustainable development of the United Nations (Lu et al. 2015), as good data are needed for designing, evaluating, and implementing political and social programs, especially in a context of aging populations and fast fertility decline that numerous countries are experiencing. Many Latin American countries are facing problems in allocating resources for their social policies, at the same time producing “out-of-date” and less reliable social indicators, given the lack of high-quality vital registration (Faijer 1994; Setel, et al. 2007).

In this paper, we focus our analysis on data quality in birth registration, using Brazil as an example for several reasons. First, Brazil is the largest country in Latin America, with a population of more than 200 million inhabitants, and very heterogeneous in terms of regions (IBGE 2018). Second, we argue that Brazil is a good example due to the improvements in data quality observed in recent years (Hunter and Sugiyama 2018), followed by a rapid fertility decline across the whole country in recent decades (Castanheira and Kohler 2017). A third reason is the availability of different data sources for estimating fertility (Cavenaghi and Alves 2016) and the existence of estimates produced by the national statistical office and other researchers.

We saw in the last three decades that birth registration has improved steadily from about 80% of registration in the 1990s to over 95% in the most recent years (Hunter and Sugiyama 2018). In addition to the improved registration of births, one can produce demographic estimates in Brazil using a variety of data sources such as the population censuses, household surveys, and Demographic Health Surveys (DHS). Thus, it is possible to derive estimates that allow to evaluate the quality of the registration system from different sources and also to produce a long-time series of fertility and mortality profiles. Nonetheless, the country is marked by high levels of heterogeneity in terms of quality of vital registration, both mortality and fertility (Queiroz et al. 2017; Hunter and Sugiyama 2018; Schmertmann et al. 2013). According to the UNICEF Global Database (2017), about 95% of the births in Brazil are registered; however, the lowest income group registers about 88% of their births, whereas the highest income group is at over 98%. In some other countries of the region, data quality is even worse, for example in Bolivia, where only 76% of the births are registered, and among the poorest income groups in that country, there is 68% under-registration of births. As we can see, defective data across countries and within countries make it difficult to harmonize and unify demographic estimates and population projections.

Usually, the estimates of fertility and mortality for many countries in the region are constructed using alternative data sources and alternative demographic methods instead of relying on vital registration statistics. This is a limitation to the development of reliable demographic estimates for the region, since even these alternative data sources have presented a number of problems linked to low coverage of population and vital events, age heaping, and age misreporting (Guzmán et al. 2006). In order to tackle these problems, numerous demographic and statistical methods have been employed and developed to assess the quality of demographic information and to provide better vital statistic estimates for the region. Most of these methods have been applied to evaluate the completeness of births and deaths, using population censuses as the main data source. In addition, they are based on strong assumptions, i.e., stable population with no migration flows, non-selectivity in age-specific rates, and these conditions are not always fulfilled, usually compromising the fertility estimates (Moultrie and Dorrington 2008; Alkema et al. 2012; Moultrie et al. 2013).

To our knowledge, there is a lack of systematic comparative analysis of the data sources and methods applied to estimate fertility indicators in the region. There are different fertility studies for various Latin American countries using differing datasets, but, in general, they do not compare sources or methods in a systematic way. In this context, the main goal of this paper is to evaluate several indirect demographic methods and data sources commonly used to estimate fertility schedules in less developed countries, and to discuss what would be best possible way to estimate fertility, given the data limitations encountered in these countries. We apply different methods and use different data sources for Brazil and its regions as a case study, given the reasons illustrated above.

In the following section, we provide a historical overview of the main data sources used to access birth information and fertility schedules in Latin America and, more specifically, in Brazil. We also present a general overview of the main demographic methods commonly applied to estimate fertility in the region, taking Brazil as a case study and using different data sources as an example. Next, we will discuss the main datasets and methods used in the region to access and correct completeness of births, namely the P/F of Brass (Brass 1964), and other less used methods such the Synthetic Relational Gompertz model (Moultrie et al. 2013) and the Own-children method. In addition to that, we will illustrate how these methods perform in the context of fertility decline and at sub-national level, using various data sources and comparing with official and unofficial estimates. Finally, we will analyze the fertility schedules of Brazil, giving central attention to the interaction between different indirect methods (described in detail in the following section) and data sources. As Brazil is the most populated country in Latin America and features a huge heterogeneity regarding socioeconomic developments and rapid demographic transitions (Saad 2011; Potter et al. 2002; Madalozzo 2012), we believe that it makes a good case study for analytical purposes.

## Brazil, a case study

Brazil is an interesting and important case study. In addition, to have a long data series on fertility, the country has gone through major demographic changes in recent years. Over the last 40 years, Brazil has experienced a rapid decline of its fertility levels (Martine 1996; Carvalho 1997; Merrick and Berquó 1983; Carvalho et al. 1981; Paiva 1987; Faria 1989; Alves 1994; Potter 1999). The total fertility rate (TFR) declined from 6.2 in 1940 to 1.8 in 2010, which implies a rate of decline of 2.5% per year. All regions in the country also observed a rapid decline in their fertility rates over this period. The data show that a faster decline was observed first in the more developed parts of the country (Southern regions). The Northeast, the poorest and less developed region, showed a slower decline in the early decades, but since 1980, the rates of decline there have been accelerating and the TFR shows some signs of convergence with the lowest ones observed in the country (IBGE 2002; Schmertmann et al. 2013).

In the 1960s, the need to obtain estimates of demographic indicators in countries with poor data quality stimulated demographers to develop indirect methods for the estimation of fertility and mortality rates. In the last five decades, many Brazilian demographers have made constant use of these methods and continue to rely on them, although the fertility and the mortality rates have already reached very low levels in some regions of the country (Cavenaghi and Alves 2016). In this context, one of the main aspects that compromise fertility data in the country is the under-enumeration or under-registration of births (often present in different data sources) which continues to impose challenges for scholars and official authorities.

## Data sources to access fertility information in Brazil

### Population censuses

The Brazilian National Statistical Office (IBGE) has a long history of data production about demographic and socioeconomic characteristics for Brazil and its regions. The most important data source is the population census, which occurs every 10 years. Censuses offer broad possibilities for population research and its demographic components, such as fertility, mortality, and internal migration.

In Brazil, as in other Latin American countries, there is a long tradition in collecting information on fertility and mortality in population censuses, even before the year 1960. In the 1940 census, the question on total (and surviving) live births to women of age 15+ was included (since 1991 to women of age 10+). In 1970, the information on children born in the previous 12 months before the survey was included. Since then, the use of such data has increased, especially in combination with indirect techniques, based on data from “children ever born” information, which allow us to estimate average parity (P), and “the number of births in the 12 months previous to the census” that produces cumulated current fertility (F), and the relationship between these two measures was used to estimate the correct level of total fertility rates (TFR) (Brass 1964; Moultrie and Dorrington 2008; Cavenaghi and Alves 2016), as well as age-specific fertility rates (ASFR) (Moultrie et al. 2013). Due to the nature of the questions asked, the scope for internal validation and cross-checking of the answers is limited. However, due to the complexity of the census enumerating every individual, it is not feasible to ask more detailed questions on fertility (Potter et al. 2002; Potter et al. 2010; Berquó and Cavenaghi 2006; Moultrie et al. 2013).

Despite being the main source of demographic information, there are many issues with census quality. For one thing, census coverage varies according to the year of the inquiry: for the four last censuses, for example, under-enumeration ranged from 1.8% in 1980 to 2.5% in 2010 (IBGE 2008; Alves 2010). Other common problems are under-reporting of children under the age of five and systematic under-enumeration of the young adult population; poor declaration of age with tendencies to reduce the age of adults, particularly among women; and tendencies to age exaggeration, especially among the 60+ population (IBGE 2008).

### Household and demographic health surveys

Household surveys are the most widespread tool for gathering social, economic, and demographic information on population. They are important tools to provide intercensal information. The current household survey system in Latin American countries dates back to the early 1960s, when many countries initiated programs designed to measure employment, unemployment, and other labor force characteristics (CEPAL 1983). Over time, additional information was collected on income, expenditure, cost of living, and living standards. This demand has also triggered the increase of survey coverage and an expansion of socioeconomic information.

As living conditions require more detailed information and variables that are not strictly related to the labor market and economic activity, these surveys started to include more detailed issues such as housing conditions and demographic variables (migration, fertility, infant mortality). In the case of Brazil, the National Household Surveys (PNAD) since 1972 have included questions related to month and year of birth of the last child born alive, and the number of children ever born, within and outside the household (IBGE 2015; Wong 1988).

Since these surveys are performed on a yearly basis, they can also serve as an up-to-date source of fertility information in the country, but a number of issues concerning sample design (Silva et al. 2002) and representativity for small aggregations prevent the use of household surveys as official source for fertility measures (Castanheira and Kohler 2015; Narita and Diaz 2016).

Demographic Health Surveys (DHS) are considered the gold standard for fertility estimation in many developing countries. They use structured questionnaires that contain detailed information of demographic events, but during the years, the range of information collected has broadened in scope to include gender issues (surveys increasingly include both men and women), HIV/AIDS, maternal mortality, violence against women, nutrition etc. In general, they characterize the female population in childbearing age and children under the age of five according to demographic, socioeconomic, and cultural factors. Patterns of marriage, parenting, and female reproduction are identified, as well as profiles of morbidity and mortality during the infancy and breastfeeding phases, and many other reproductive and health aspects (DHS 2015; PNDS 2006).

Despite their limitations, the full birth histories have become the dominant source of estimates of fertility levels and trends for countries lacking complete birth registration (Avery et al. 2013). These reproductive health reports usually present very detailed information about the date of birth of each child for all women and very useful material for studying fertility levels and their cohort trends. In addition, DHS data historically have played an important role in countries such as Colombia, Peru, and Haiti where, due to lack of trustworthy information from censuses or civil registers, the official fertility estimates rely mainly on DHS information (Di Cesare and Rodriguez Vignoli 2006; Departamento Administrativo Nacional de Estadistica – DANE 2000; DHS 2015).

In Brazil, the first DHS was performed in 1986 and was later renamed to PNDS (2006) (Pesquisa Nacional de Demografia e Saúde da Criança e da Mulher), a survey that follows the same questionnaire structure and presents the same amount of information as the Demographic Health Surveys (Castanheira and Kohler 2015; Tejada et al. 2017).

### Vital registration systems

In many Latin American countries, the quality of vital statistics varies through time (Mikkelsen et al. 2015). Coverage has improved in some countries, whereas in others, registration of births and deaths is still rather incomplete (Guzmán et al. 2006). Generally, birth registration is of better quality than death registration, but neither appears to be improving much in countries where quality is poor, for example, in Peru, Bolivia, Ecuador, the Dominican Republic, and Venezuela (Duryea et al. 2006; Lima and Queiroz 2014; PLoS Medicine Editors 2010; Setel, et al. 2007; AbouZahr and Boerma 2005). In these countries, registration of births is incomplete, because parents often lack incentives to register or because babies who die shortly after birth may be registered neither as births nor deaths. Late registration of births also occurs very often in the region. This may mean that there is a delay of several years before all the children from a given birth year cohort are registered, for example, when the child attains schooling age (Moultrie et al. 2013). On the other hand, in some countries like Chile, the proportion of late-registered births declined steadily from 10 to 15% before 1980 to 5% in the late 1980s and 1990s and further to around 1% in 2001–2004 (Zeman and Castro 2014).

In 1989, the newly formed Vital Statistics Group of the Ministry of Health created an information system of its own for collecting information on live births in Brazil, which duplicated the task already carried out by the National Statistical Office (Mello Jorge et al. 2007). Since then, the Ministry of Health has provided annually births information through the System of Birth Information (SINASC), which aims to supply all spheres of the health system with reproductive information about the whole Brazilian population. The system includes consolidated data since 1994, and its information is based on the standardized model of the Live Births declaration (Frias et al. 2014).

The SINASC has progressed consistently (Mello Jorge et al. 2007), and many studies indicate that recent coverage of births is higher than 90% at country level (Pedraza 2012; Hunter and Sugiyama 2018). However, looking at more disaggregated units, under-registration of births still varies considerably across regions of the country. Considering other variables, like mother’s educational level, race, and number of prior childbirths, the inconsistency in information is even higher (de Oliveira et al. 2015 and Hunter and Sugiyama 2018).

## Methods

### Brass P/F ratio adjustment (one-census method)

According to Brass (1964), data on current fertility in demographic censuses (or household surveys) are generally underestimated for all age groups, and some empirical evidence showed that this underestimation does not differ by the age of women. This fact led to an adjustment factor for TFR, estimated by the comparison of cumulated fertility (*F*) from a period with the average parity distribution (*P*), measurement of cohort fertility (United Nations 1983). Brass defined *P* as the average parity (cumulated lifetime fertility) of a cohort of women up to a given age and *F* to be closely related to the cumulated current (period) fertility up to that age. The P/F ratio expresses relation of these two quantities for each age group (Brass 1964; Moultrie and Dorrington 2008).

The method relies on the fact that if a fertility schedule has remained constant for an extended period of time, the cohort and period measures should be identical. In other words, under conditions of population stability, with constant fertility and mortality rates, the cumulated fertility of a cohort of women up to any given age will be the same as the cumulated fertility up to that same age in any given period, and if the data has no errors, the P/F ratio would equal to 1 in every age group (Brass 1964; United Nations 1983; Moultrie and Dorrington 2008; Baker et al. 2011; Hauer et al. 2013).

The method also assumes that the fertility of the women who do not survive is not very different from the surviving women. This means that the surviving women do not show significantly different levels of childbearing than women already deceased, and the cumulated fertility of a cohort of women up to any given age is the same as the average parity in that cohort (Brass 1964; United Nations 1983; Moultrie and Dorrington 2008; Baker et al. 2011; Hauer et al. 2013). However, according to Moultrie et al. (2013), this last assumption is not all that important as in most modern populations, the magnitude of female mortality in the reproductive ages is very small and the effect of differential survival will therefore be negligible.

When fertility is falling, the cumulated lifetime fertility (*P*) would be greater than cumulated current fertility (*F*). In this case (and in the absence of errors in the data), the P/F ratio would depart from unity systematically with increasing age of mothers. We would expect the P/F ratio to be fairly close to unity at the youngest ages, because even by women’s mid-twenties, one would not suppose any significant deviation of cumulated period fertility from cumulated lifetime cohort fertility, as most of the births to women in that cohort would have happened fairly recently.

In this case, the P/F ratio derived from women aged 20–24 at the time of a survey is held to be the most reliable indicator of the quality of fertility data thus collected. Conveniently, the supposition is that the average parities of younger women are usually fairly accurately reported, at least in comparison with those of older women (Moultrie et al. 2013; United Nations 1983). Cases of strong fertility decline associated with variations in the level and pattern of current fertility might affect the method, presenting an error of 5% to the adjusted TFR (Moultrie and Dorrington 2008). In order to avoid such an error, other methods have been implemented, commonly known as extensions to Brass P/F ratio, but based on the estimation of age-specific fertility from the increment of cohort parities between two censuses (United Nations 1983).

Moultrie and Dorrington (2008) discussed other limitations and alternatives to the Brass P/F method, showing several setbacks of that method in different scenarios. The main limitation that they point out is the changes in fertility levels and changing age distribution of fertility. They provide simple adjustment to the Brass method. This adjustment uses information from two censuses (Arriaga 1983; United Nations 1983), and in this case, the assumption of constant fertility can be relaxed. Thus, the cumulated current fertility can be compared to the cumulated cohort parity increments from one data point to the other. Another adjustment method, proposed by Feeney (Feeney 1998), uses mean ages of childbearing as the points to consider for adjusting the fertility levels by the P/F ratio.

### The Synthetic Relational Gompertz model (two-censuses method)

The Synthetic Relational Gompertz (SRG) model is a conceptual improvement of the Brass P/F method and an extension of the relational Gompertz^1^ method for the estimation of age-specific as well as total fertility rates. This model makes use of two sets of parity data, collected at different points in time, in combination with estimates of current fertility in the intervening period based on reports of recent births classified by age (Moultrie et al. 2013; Booth 1984; Zaba 1981).

In concrete terms, the Gompertz relation assumes that the proportion of accumulated fertility follows a linear distribution of the following equation:

1 $$ \frac{F(x)}{\mathrm{TFR}}={e}^{\left(\alpha \times {e}^{\left(\beta \right)}\right)} $$

where *F* (*x*) is the accumulated fertility up to age *x*, TFR the total fertility, and α and β are two parameters. This function can be reduced to a linear form based on the following log transformation:

2 $$ \ln \Big(-\ln \left(\raisebox{1ex}{$F(x)$}\!\left/ \!\raisebox{-1ex}{$\mathrm{TFR}$}\right.\right)=\ln \left(-\alpha \right)+\beta x $$

In this sense, we use an equation that is quite similar to the Brass logit, because the purpose of both is to perform a linear transformation of a distribution so that it can be compared to a standard distribution using two constants, *α* and *β*. In this model, *α* is an indicator of the location of the fertility pattern in relation to age, while *β* is interpreted as the determinant of the dispersion or degree of concentration of the fertility pattern. In short, we can say that a negative value of *α* means a curve that is more inclined to the right while a positive one indicates a more left-skewed curve. With respect to *β*, a value greater than 1 is represented by a narrower curve and a value smaller than 1 by a wider curve.

The relational Gompertz method seeks to correct the errors commonly found in fertility data associated with too few or too many births being reported in the reference period and the under-reporting of lifetime fertility and errors of age reporting among older women. In sum, the Gompertz method has the additional advantage of smoothing age-specific fertility rates, a major issue when dealing with sub-national populations, and reduces potential problems of reporting errors in parity by older women. One important aspect is that the method requires some knowledge of the demographic patterns in the study area (Moultrie and Dorrington 2008).

The extension of this method, the Synthetic Relational Gompertz, allows changes in fertility to be taken into account and is designed to be applied to censuses or surveys conducted either 5 or 10 years apart (Moultrie et al. 2013; United Nations 1983). In such circumstances, the change in the average parity of the cohort can be estimated, as the survivors of a cohort of women at the first census can be identified at the second one. By cumulating the sequence of parity increments for different cohorts during the period between the censuses, we estimate average parities for a hypothetical cohort experiencing the fertility implied by the observed parity increments (Moultrie et al. 2013). The period fertility rates that are compared with these synthetic cohort estimates should ideally refer to the entire period between the two censuses that asked about lifetime fertility. This comparison gives the true estimate of TFR for the period in question (Moultrie et al. 2013). Because the Synthetic Relational Gompertz model uses information from two censuses or household surveys, any improvement or change in data quality over time between the two inquiries may also affect fertility pattern and estimates. Also, any relational model relies on the standard used, and serious deviations between the observed information and the standard function might also affect the estimates of fertility (Zaba 1981).

### The Own-children method and the reconstruction of fertility history

The Own-children method (OCM) is a census- or survey-based reverse survival technique designed to estimate age-specific fertility for the period preceding the enumeration (Retherford and Cho 1978). In the absence of detailed data on reproduction, this reverse survival method, also called reverse projection, uses the current age structure and assumptions about mortality in a given population to reconstruct the age structure of this population in the period preceding the survey.

The estimation follows an explicit order. First, the enumerated children are matched to their mothers living in the same household (Retherford and Cho 1978; Cho et al. 1986). These matched (i.e., their “own”) children, classified by their own age and their mother’s age, are “reverse-survived” to estimate the number of births by age of mother in previous years. Reverse survival is similarly used to estimate the number of women by age in the past. After adjustments for under-enumeration (mainly due to undercount and age misreporting) and unmatched (i.e., their “non-own”) children, age-specific birth rates are calculated by dividing the number of reverse-survived births by the number of reverse-survived women (Retherford and Cho 1978; Avery et al. 2013).

The allocation of children (until 14 years of age) to the respective mother (from 15 to 64 years of age) in the census data is based on the information about their relationship to the head of household, to other women in the household, and the mothers’ information on the number of children ever born and children surviving. Once the data were matched, we tabulated age of children by age of their mothers. In some cases, it was not possible to match children with their mothers. This occurs either because the information about the relationship to the head of the household is insufficient or because some children do not live in the same household as their mothers or because the mother has deceased. These children are so-called “other than own” or simply “not-own” children in the household. They are proportionally distributed by age of mother using the age distribution of the mothers with identified children of their own. Besides, this addition of “not-own” children, age misreporting, and age under-enumeration can bias the data as well. Finally, a survivorship function is applied to generate the number of births by age of mother for the years prior to the survey (Retherford and Cho 1978; Avery et al. 2013).

The OCM has important advantages over other methods that reconstruct age-specific fertility rates from census or survey data (Retherford and Cho 1978; Avery et al. 2013). The method can simply be applied to an existing census or survey, and it does not require any additional data, except for life tables or mortality estimates used in computing reverse survival. If these are not available, survivorship may also be estimated directly from the census or survey by linking estimates of child mortality—based on commonly asked questions regarding the total numbers of children ever born and children surviving—to appropriate model life tables. Furthermore, the OCM is not very sensitive to life table estimation errors under mortality levels currently prevailing in most parts of the world (Retherford and Cho 1978; Cho et al. 1986). The results of these estimates are available on the website of the Human Fertility Collection (HFC 2017; www.fertilitydata.org). The HFC web page there gives series of fertility rates for Brazil as a whole, estimated for the years 1966 to 2010 (Lima 2013).

## Results

For the first analysis, we make use of data from population censuses 1970 to 2010. We tabulate the information on the number of children ever born classified by 5-year age groups of mothers, taken from each census; the number of births during the year preceding each census, classified by 5-year age groups of mothers; and the number of women in each age group. These tabulations are made for the urban Rio Grande do Norte (RN) State. Rio Grande do Norte is one of the smallest states in Brazil and has been going through rapid changes in fertility. We used this sub-population as example for two main reasons: first, because Rio Grande do Norte is located in the Northern region of the country, a part of the country historically known for having the worst data quality (IBGE 2003; Paes 2006; Lima and Queiroz 2014). Second, the demographic transition process, together with changes in levels and shape of fertility has been more profound than in any other region of the country; therefore, the assumptions of the demographic methods can be better evaluated (IDEMA 2002; Fossa and Bezerra 2002).

Further tabulations are also made for the country as a whole, but only for census years 2000 and 2010. We make use of birth registers publicly available by the Ministry of Health Database (DATASUS) and other official estimates provided by the National Statistical Office (IBGE), the Human Fertility Collection (HFC 2017) and PNDS (2006). This time, our concern is to compare the results of different data sources and methods in a scenario of fertility decline to below-replacement level.

### Comparison between traditional Brass P/F method and Synthetic Relational Gompertz model in a scenario of fast fertility change

**Fig. 1:**
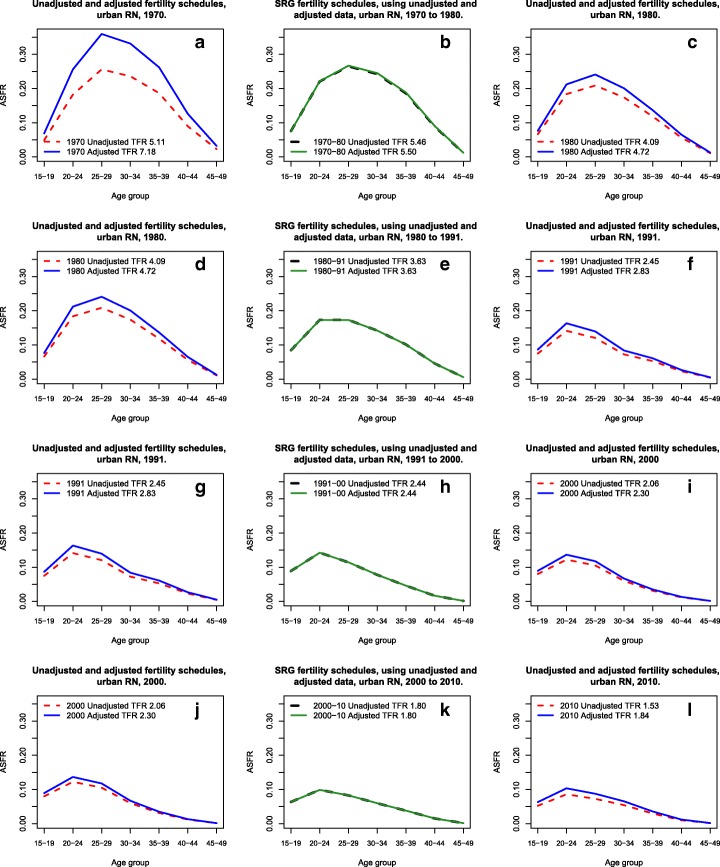
Fertility schedules estimated by traditional Brass method and by SRG model, urban Rio Grande do Norte (RN), 1970 to 2010. Source: Brazilian censuses 1970–2010

Figure 1a–l shows the performance of the P/F adjustment versus the Synthetic Relational Gompertz in the context of fast-falling fertility development. Here, we analyze the case of the urban sub-population of the Rio Grande do Norte (RN) state. This region has experienced a sharp fertility decline between the years 1970 and 2010, with an observed TFR of 5.11 in 1970 and 1.53 in 2010, representing a fertility decrease of almost 70% in a 40-year period.

For the test of methods performance, each time we created two groups of estimates, one without correction, i.e., the observed data extracted directly from the censuses, and second with level correction in age-specific fertility rates, given by P/F ratio from the 20–24 age group. Apart from that, fertility schedules were estimated between censuses, using the Synthetic Relational Gompertz model, without and with P/F ratio adjustment.

From 1970 to 1980, Fig. 1a–c, with the exception of the group of 15–19 years of age, we observed a sharp decline in fertility, especially after the age of 25. The P/F adjustment raised the level of fertility to a TFR of 7.18 (an increase of approx. 41% in the ASFR) in 1970 and to 4.72 (15% increase in the ASFR) in 1980. While with the SRG estimates, we see no significant changes in the pattern nor in the level of fertility, before and after the Brass correction was applied, e.g., the SRG models give a TFR ranging from 5.46 to 5.50 (with and without correction, respectively). The values are very similar, showing a good consistency of the method in a scenario of fast fertility change:

1. TFR 5.11 (1970) ⟹ 5.46 (1975) ⟹ 4.09 (1980) ⟹⟹Unadjusted data
2. TFR 7.18 ⟹⟹⟹⟹5.50 ⟹⟹⟹⟹ 4.72 ⟹⟹⟹⟹ P/F adjusted data

Taking into account that past fertility was considerably higher in the State of Rio Grande do Norte and falling rapidly, one can argue that P/F adjusted data is more plausible.

In the second period, Fig. 1d–f, between 1980 and 1991, the decline in fertility was more pronounced and faster than in the previous one (about 40% fertility decline in that period), and it also occurred in almost all age groups (again with exception of the 15–19-year-olds who showed a slight increase in their specific rates). In 1991, the adjustment P/F was 16%, raising the TFR to 2.83.

The TFR estimated for the intercensal period by the SRG did not change in value (TFR of 3.63). The same can be said about the pattern of fertility schedules, which practically overlapped. Moreover, once again, we have two scenarios of fertility change to choose.

1. TFR 4.09 (1980) ⟹ 3.63(1986) ⟹ 2.45 (1991) ⟹⟹Unadjusted data
2. TFR 4.72 ⟹⟹⟹⟹ 3.63 ⟹⟹⟹ 2.83 ⟹⟹⟹⟹⟹P/F adjusted data

Since we did not observe any changes in both pattern and fertility level estimated by SRG, we take this estimate (middle year between censuses) as a benchmark for comparison. In the first scenario, when we analyze the first 5-year period, fertility fell by almost 11% and by 33% from 1980 to 1991. The second scenario shows a fertility drop of 23% (between 1986 and 1986) and 22% (between 1986 and 1991). Despite small differences, it is more plausible that the biggest drop occurred in 1980–1986 (scenario 2), while we expected the strongest fertility declines to have taken place in the past, when fertility levels were higher.

From 1991 to 2000, Fig. 1g–i, the decline in fertility was less pronounced (16–19% during the period, considering the observed data or the ones corrected by the Brass method). The SRG estimates continued to have similar values, with a TFR of 2.44 (in 1996). The fertility pattern was also very similar between estimates. This fact might also indicate the robustness of the method.

1. TFR 2.45 (1991) ⟹ 2.44 (1996) ⟹ 2.06 (2000) ⟹⟹Unadjusted data
2. TFR 2.83 ⟹⟹ ⟹ 2.44 ⟹⟹⟹ 2.30 ⟹⟹⟹⟹ P/F adjusted data

Both possibilities are plausible, but it is more likely to believe that from 1991 to 1996, fertility dropped instead of remaining stable. Therefore, we disregard the first scenario, without P/F ratio correction.

For the last period of analysis 2000 to 2010, Fig. 1j–l, the decline in fertility was extended to adolescent and young adult ages (under 30 years). The P/F adjustment indicates an increase in the under-enumeration of births of 20%. This is something that is contrary to expectations, i.e., a continued improvement in data collection over the years in the country. However, the effect of the sharp drop in teenage fertility in Brazil between 2000 and 2010 may be affecting the estimates of P/F ratio (Carvalho et al. 2016; Castanheira and Kohler 2015). Moreover, this effect does not seem to affect the relational Synthetic Gompertz model which continues to show very similar fertility schedules in both scenarios considered.

1. TFR 2.06 (2000) ⟹ 1.80 (2005) ⟹ 1.53 (2010) ⟹⟹Unadjusted data
2. TFR 2.30 ⟹⟹ ⟹ 1.80 ⟹⟹⟹ 1.84 ⟹⟹⟹⟹ P/F adjusted data

Unlike in previous analyses, the second scenario with fertility correction in 2010 seems less plausible: first, because the likelihood that fertility has increased between 2005 and 2010 is rather low, and second because it is hard to believe that the Brass method is reliable in this case. As a first sign, the P/F of Brass seems to work fine even when the condition of stability is violated. However, if the decline of fertility affects younger ages, the method leads to misleading level correction. On the other hand, the SRG model gives more robust results, independent from the scenario chosen.

### Comparison between data sources and Brass, Synthetic Relational Gompertz, and Own-children method

**Fig. 2:**
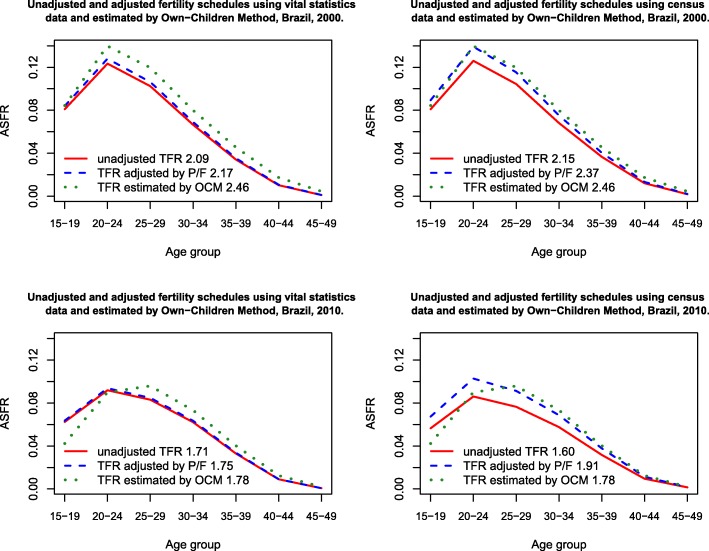
Fertility schedules estimated by different methods and different data sources, Brazil, 2000 to 2010. Source: Brazilian censuses and vital registration 2000–2010

In this section, we compare both methods with the estimates generated by the Own-children method, but this time using different data sources, i.e., information derived from the civil registration and censuses for Brazil as a whole. Table 1 and Fig. 2 show the estimates while we take as sources the civil registration versus census data for the years 2000 and 2010 and other official and unofficial estimates.

**Table 1:** Comparison between TFRs according to different data sources and estimates, Brazil, 2000–2010

Source of data	Year of the inquiry			Plausibility
	2000	2005	2010	
Vital registration	Unadjusted TFR	SRG-estimated TFR	Unadjusted TFR	Plausible
	2.09	1.75*	1.72	
	P/F-adjusted TFR		P/F-adjusted TFR	Plausible
	2.17	1.75*	1.75	
	Official estimates (IDB SINASC/DATASUS, 2013)			
	2.29	1.99	1.82	
Census	Unadjusted TFR	SRG-estimated TFR	Unadjusted TFR	Plausible
	2.15	1.76*	1.60	
	P/F-adjusted TFR		P/F-adjusted TFR	Less plausible
	2.37	1.76*	1.91	
	Own-children method			
	2.46	1.96	1.78	
	Official estimates (IBGE 2012)			
	2.38	−	1.90	
PNDS		1.80		

Sources: DATASUS 2000 and 2010 and Censuses – IBGE, 2000 and 2010

Note: P/F correction applied, 4% increase in the ASRFs in 2000 and 2% in 2010, for vital registration data. For census data correction increment ASRF of 11% in 2000 and 19% in 2010

Official DATASUS estimates based on direct and indirect methods

SINASC/DATASUS official data provided by Indicadores e Dados Básicos – Brazil –IDB (2013)

*Synthetic Relational Gompertz intercensal estimates. Own-children method estimates provided by HFC (2017), estimated by Lima (2013)

The fertility schedules differ slightly between the sources. However, the adjustment by the Brass method generates different estimates, according to the source used. The civil records always present lower fertility levels than census data, and the P/F ratio also points to small corrections necessary (4% in 2000 and 2% in 2010), to civil registration data (Cavenaghi and Alves 2016). Considering the census data, the TFRs presented the highest level in 2000, but in 2010, fertility declined to 1.60 (unadjusted data). The Brass correction is also more pronounced for census data (indicating under-enumeration of births on the order of 19%), increasing the TFR to 1.91 in 2010 (Cavenaghi and Alves 2016). This is something unexpected and unlikely to happen, and further research needs to be conducted.

As mentioned by Castanheira and Kohler (2015), in Brazil, the usually TFR adjustment used is the P2/F2 ratio, which is the total parity in the age group 20–24 (P2) in relation to the cumulated period fertility (F2) in the same age group. This ratio is multiplied by the TFR or age-specific fertility rates, increasing or decreasing its level. The Brass method is very sensitive to the age group choice. Traditionally, our official fertility estimates are based on Brass method correction; however, this adjustment is also sensitive to the assumption of stable population and constant fertility, which was not the case in the country in the last 50 years. In addition to the violation to this assumption, P2/F2 ratio might be overestimating the TFR; as also mentioned by Castanheira and Kohler (2015), the TFR could be overestimated by 8% in 2010.

Regardless of the data source, the comparison between Brass and OCM shows that for 2000, both methods produce relatively similar estimates, with only minor differences at older ages of the reproductive period (see Fig. 2). The level of fertility, after correction, was also quite similar (using Brass adjustment for census-based TFR of 2.37 vs. 2.46 based on OCM estimates). This is expected since the OCM estimates and the rates (based on the information on births in the twelve months prior to the survey) are from the same data source. Considering the estimates with civil registers as a source, we again notice that fertility shows a relatively similar structure, but with tiny differences in levels. In both cases, the differences at younger ages are relatively small, especially for the civil records.

However, despite the small differences in level, in 2010, the fertility structure differs considerably between methods, and the main differences are encountered at the young ages (15–19 years) with civil records. However, when taking into account census data, there are differences in virtually all ages. The estimates for the OCM show a fertility peak at the age group 25–29, whereas estimates based on other sources still present the 20–24 age group at the highest reproductive level (Fig. 2).

It is important to highlight that the OCM has several methodological limitations. The first one is related to distortions in the age pattern of fertility and the estimated fertility trend, caused by age misreporting. Age misreporting typically causes year-to-year fluctuations in own-children fertility estimates. Another drawback relates to the method itself. The OCM does not work with complete childbearing histories, since the method does not take into account children who, at the time of the census, were dead or not present in the household. Finally, the constraint of allocation errors must be considered, concerning “own children” and “not-own children,” even if this bias tends to be less serious than age misreporting (Cho et al. 1986). However, according to Avery et al. (2013), all these problems affect the Own-children method only to a limited degree.

**Fig. 3:**
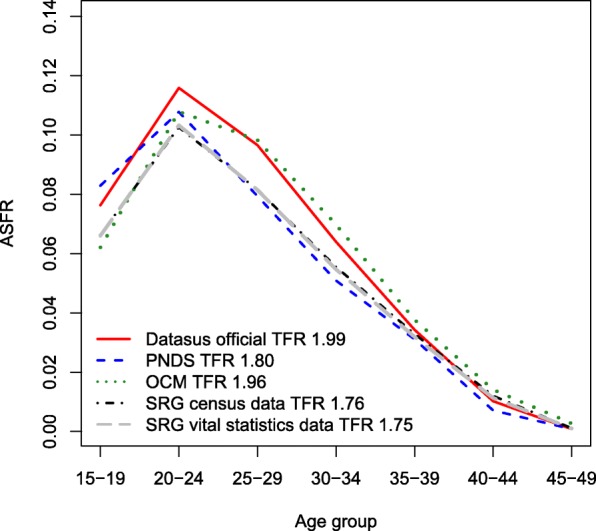
Fertility schedules estimated by different methods and different data sources, Brazil, 2005. Source: PNDS 2006 and Brazilian censuses and vital registration 2000–2010

Figure 3 shows the estimates of fertility schedules for the period of 2005, comparing how different sources and methods of fertility estimation perform in combination. The SRG model estimates with and without P/F adjustment are quite similar; therefore, we show the results for unadjusted data only.

The PNDS (2006) estimates correspond to a reproductive period of approximately 36 months before the survey, e.g., it produces estimates for the period 2004–2006. Own-children method estimates refer to year 2005, and the official estimates provided by the Ministry of Health (DATASUS) use direct and indirect methods to obtain fertility schedules (IDB 2013).

On the one hand, we did not observe distinctive differences between the estimates generated by the Synthetic Relational Gompertz model. This is true regardless of the data source (whether it is census or vital statistics), and whether or not P/F corrections are applied to fertility levels, which may be an indication of the robustness of the method. In addition to that, the average parity, contained in the synthetic cohort and estimated by the SRG, seems to capture quite well the reproductive changes during the intercensal period, just as the model appropriately confines the pattern inherent in these rates.

On the other hand, even with similarity in fertility level, the PNDS (2006) shows a large difference in terms of structure, indicating a high level of teenage fertility from 2004 to 2006, compared with the SRG model, though the fertility among other age groups is clearly slightly underestimated. Furthermore, the Own-children method also appears to generate small over-estimations of fertility rates between the ages of 25 to 30 years, if we compare to the SRG estimates, but is more similar to official numbers from DATASUS. This is also reflected in the TFR of 1.96 and 1.99, which are very close to the level of population replacement, while the other estimates are somewhat below this level.

## Conclusion

In this paper, we have provided an overview of the data sources and methods used to estimate fertility, level, and age-profile, in developing countries, using Brazil as a case study. Additionally, in the absence of high-quality vital registration systems, census data and household surveys, such as DHS, are the most common sources of information to investigate the evolution of fertility and its differentials.

Vital registration systems are the most desirable source for demographic analysis, since they usually contain up-to-date information and have a broad geographical coverage. However, the majority of countries in Latin America, including Brazil, are still lacking a well-structured institutional system for collecting such information (Guzmán et al. 2006; Faijer 1994). Furthermore, lack of periodicity and under-registration errors are common shortcomings (United Nations 2015), making this source less accessible when it comes to providing reliable fertility information. Hence, official TFR estimates in many countries, such as Brazil, are obtained from population censuses and Demographic Health Surveys or national household surveys, depending on their availability (Faijer 1994; Castanheira and Kohler 2015; Carvalho et al. 2016; Sacco and Borges 2016; World Health Organization 2016).

As data quality and availability are limited in Brazil just as in other Latin American countries, it is necessary to apply demographic methods to obtain robust estimates. We applied some of these methods: (1) P/F ratio of Brass, (2) Synthetic Relational Gompertz models, and (3) Own-children method of retrospective reconstruction of fertility schedules. We perform a series of analysis using these methods and investigate how they perform, considering different data sources and demographic circumstances. There is a wide variation in estimates as they require specific conditions, i.e., stable population, which cannot always be fulfilled, especially in the context of rapid fertility decline. We show and argue that Brass P/F potentially overestimates the country’s TFR in recent periods, as also observed by Castanheira and Kohler (2015).

Different data sources yield different results, giving a significant variability in TFRs and ASFRs. They can vary somewhat from above- to below-replacement level and present different age schedules, depending on the method and the data. Insofar, we conclude that these estimates can be very misleading, especially in the context of sub-replacement fertility that some countries in the region have been facing recently (Lima et al. 2018), or in the scenario of persistently high levels of teenage motherhood. Most important, the fertility of 15–19-year-old women varies enormously when we compare different sources and methods. These findings have considerable implications, in particular when discussing public policies aimed at increasing fertility to replacement level and campaigns to reduce teenage pregnancy, because we do not know the “true” level and shape of fertility.

Concerning the methods, we argue that the combination of the Synthetic Relational Gompertz model and Brass P/F ratio is a good tool for evaluating completeness of births and estimating the “right” fertility shape, especially in the scenario of fast fertility change. However, we argue that Brass P/F has some serious limitations and should only be used in very specific contexts, when additional data points are not available (see also Castanheira and Kohler 2015; and Moultrie and Dorrington 2008). We demonstrate that the Synthetic Relational Gompertz gives the most robust results, independent of the source used, since it allows for changes in fertility to be taken into account. However, this method also has its limitations, e.g., one has to be careful with changes of birth data quality between the two censuses, since improved quality will affect the pattern of fertility (Zlotnik and Hill 1981).

As future guidelines, we argue for the importance to improve birth registration in the region. It is important to invest and promote more accurate information about age of mother, place of residence of the mother, and further birth information including socioeconomic characteristics of the father or mother (Tacla 2009). It is also important that population data, from censuses or other estimates, is of good quality. This requires greater investments in vital records systems as well as more campaigns to inform people about the importance to properly declare births.

## Additional file

Additional file 1: R codes to produce estimates and figures presented in the paper. (R 14 kb)

## Footnotes

1 The relational Gompertz method is a refinement of the Brass P/F ratio method that seeks to estimate age-specific and total fertility by determining the shape of the fertility schedule from data on recent births reported in censuses or surveys, while determining its level from the reported average parities of younger women.
